# A convenient machine learning model to predict full stomach and evaluate the safety and comfort improvements of preoperative oral carbohydrate in patients undergoing elective painless gastrointestinal endoscopy

**DOI:** 10.1080/07853890.2023.2292778

**Published:** 2023-12-18

**Authors:** Yuzhan Jin, Mingtao Ma, Yuqing Yan, Yaoyi Guo, Yue Feng, Chen Chen, Yi Zhong, Kaizong Huang, Huaming Xia, Yan Libo, Yanna Si, Jianjun Zou

**Affiliations:** a School of Basic Medicine and Clinical Pharmacy, China Pharmaceutical University, Nanjing, China; b Department of Clinical Pharmacology, Nanjing First Hospital, Nanjing Medical University, Nanjing, China; c Department of Anesthesiology, Perioperative and Pain Medicine, Nanjing First Hospital, Nanjing Medical University, Nanjing, China; d Department of Anesthesiology, Leping People’s Hospital, Jiangxi, China; e Department of Pharmacy, Nanjing First Hospital, China Pharmaceutical University, Nanjing, China; f Nanjing Xiaheng Network System Co., Ltd., Nanjing, China; g Jiangsu Kaiyuan Pharmaceutical Co., Ltd., Nanjing, China

**Keywords:** Full stomach, aspiration, machine learning, propensity score matching, preoperative oral carbohydrates, gastrointestinal endoscopy

## Abstract

**Background and aims:**

Assessment of the patient’s gastric contents is the key to avoiding aspiration incidents, however, there is no effective method to determine whether elective painless gastrointestinal endoscopy (GIE) patients have a full stomach or an empty stomach. And previous studies have shown that preoperative oral carbohydrates (POCs) can improve the discomfort induced by fasting, but there are different perspectives on their safety. This study aimed to develop a convenient, accurate machine learning (ML) model to predict full stomach. And based on the model outcomes, evaluate the safety and comfort improvements of POCs in empty- and full stomach groups.

**Methods:**

We enrolled 1386 painless GIE patients between October 2022 and January 2023 in Nanjing First Hospital, and 1090 patients without POCs were used to construct five different ML models to identify full stomach. The metrics of discrimination and calibration validated the robustness of the models. For the best-performance model, we further interpreted it through SHapley Additive exPlanations (SHAP) and constructed a web calculator to facilitate clinical use. We evaluated the safety and comfort improvements of POCs by propensity score matching (PSM) in the two groups, respectively.

**Results:**

Random Forest (RF) model showed the greatest discrimination with the area under the receiver operating characteristic curve (AUROC) 0.837 [95% confidence interval (CI): 79.1–88.2], F1 71.5%, and best calibration with a Brier score of 15.2%. The web calculator can be visited at https://medication.shinyapps.io/RF_model/. PSM results demonstrated that POCs significantly reduced the full stomach incident in empty stomach group (*p* < 0.05), but no differences in full stomach group (*p* > 0.05). Comfort improved in both groups and was more significant in empty stomach group.

**Conclusions:**

The developed convenient RF model predicted full stomach with high accuracy and interpretability. POCs were safe and comfortably improved in both groups, with more benefit in empty stomach group. These findings may guide the patients’ gastrointestinal preparation.

## KEY MESSAGES

This study is the first model utilizing advanced ML techniques based on multiple clinical variables to identify full stomach. The model is suitable for patient-rich outpatient clinics, primary hospitals, remote regions, and specific clinical settings where POCUS is not available.

The developed convenient RF model predicted full stomach with high accuracy and interpretability. The test cohort AUROC was 0.837. We further established an online accessible individualized risk calculator and provided waterfall plots to increase the interpretability of each prediction.

The propensity score matching (PSM) showed that preoperative oral carbohydrate (POCs) were safe and comfortably improved in both groups, with more benefit in empty stomach group. These findings may provide information for anesthesiologists to guide patients on POCs.

## Introduction

Gastrointestinal endoscopy (GIE) is the gold standard for screening many gastrointestinal diseases, and for better tolerance, most GIE procedures are sedated [1–4]. Increased gastric contents may lead to pulmonary aspiration during sedation, the single most common cause of death associated with airway management [5]. Aspiration mostly occurs in emergency cases, and empty stomach can be achieved by fasting in elective surgical patients [6]. However, fasting is not completely reliable, especially when patients with delayed gastric emptying diseases may still be full despite fasting [7]. It has been reported that the incidence of full stomach in routinely fasted elective patients is 33.7% [8]. Therefore, it is necessary to identify the full stomach ahead of GIE sedation, which is the key to avoiding aspiration incidents.

Gastric point-of-care ultrasound (POCUS) is a practical imaging method for describing gastric contents and volume [9]. Perlas et al. proposed a POCUS-based 3-point grading system for semi-quantitative assessment of gastric contents, and it was validated in later studies [10,11]. Several linear models based on the antral cross-sectional area (CSA) were developed to assess gastric fluid volume quantitatively [8,12–14]. However, they are limited in efficiency and require POCUS devices and skilled operators, making them difficult to popularize in patient-rich outpatient clinics, especially in primary hospitals and remote regions. And these studies were only based on traditional linear analysis methods, which have room for improvement in accuracy [15]. In addition, these studies did not adequately investigate the patients’ clinical information and medical history, and substantial GIE patients combined these risk factors and presented with delayed gastric emptying [7,16].

In contrast, machine learning (ML) algorithms, part of artificial intelligence, are based on advanced statistical techniques to unravel the complex relationships between numerous variables and can make predictions as accurately and quickly as possible, relying on large amounts of data [17]. ML models have been increasingly mentioned in personalized medicine, including the diagnosis and risk prediction of gastrointestinal diseases [18,19]. Its convenience and accuracy may help to overcome the limitations of POCUS. Therefore, we build an ML model to discriminate patients with empty- or full stomachs.

In addition, current gastric contents assessment methods [8,10–14] focus only on immediate outcomes and do not guide patients’ gastrointestinal preparation. As a new paradigm of preoperative gastrointestinal preparation, preoperative oral carbohydrates (POCs) can improve the discomfort induced by fasting, but there are different perspectives on the risk of full stomach. Some studies showed that POCs do not increase or even decrease the patient’s gastric fluid volume [20–22]. Most recent practice guidelines also recommend that healthy patients take clear liquids two hours before surgery to relieve discomfort caused by excessive fasting [23,24]. However, a study has shown that POCs 2h before surgery in cirrhotic patients may increase the risk of regurgitation [25]. Therefore, with the model outcomes, evaluating the safety and comfort improvement of POCs in different stomach risk groups could guide patients in using POCs.

In this study, we aim to develop the most convenient ML model to predict full stomach in the Chinese elective GIE cohort and evaluate patients’ safety and comfort improvements after POCs. We are dedicated to accurate prediction outcomes that will prompt anesthesiologists to pay close attention to the full stomach patients and precise the patients’ gastrointestinal preparation.

## Methods

### Study subjects

The study protocol complies with the declaration of Helsinki and received approval from the Nanjing First Hospital medical ethics (No. KY20220518-05-KS-01). The requirements for informed consent were waived by the ethics committee as the study was retrospective. This study follows the TRIPOD guidelines for the prediction model [26].

The study was performed in Nanjing First Hospital, China. Between October 2022 and January 2023, all GIE patients who satisfied the painless GIE standard procedure requirements were prospectively recruited in this endoscopy institution, and we retrospectively collected the records of these consecutive GIE patients from the electronic medical system. In our research, the inclusion criteria were as follows: (1) patients were adults (age 18 years old or older); (2) American Society of Anesthesiologists physical status (ASA PS) I, II, III. Those who satisfied painless GIE procedure requirements would be adequately informed of the fasting requirements, including the reasons to fast for at least 8 h and refrain from drinking for at least 4 h. Patients undergoing painless colonoscopy or with missing data (including POCUS data or Visual Analogue Scale (VAS) score) were excluded from the analysis.

### Data collection

In this research, we provided POCs according to the clinical diagnostic procedure, patients were in the POCs group if they used POCs before anesthesia and in the non-POCs group if they did not. GIE patients who received POCs were in the POCs group, and those without POCs were in the control group. The control group was divided into training and testing cohorts to construct and validate the model of predicting ‘full stomach’. For the POCs group, patients should drink 200 ml of 12.5% carbohydrate until 3 h before the elective painless GIE procedure. For the control group, patients would be completely fast.

All clinical data, POCUS information, and VAS score were collected by experienced researchers using a structured form. Potential predictors of full stomach were chosen from the previous studies, including basic characteristics (Age, BMI, Gender, Drinking, Smoking), preoperative management data (Fasting time, No drinking time, Preoperative diet), clinical information (Asthma, Parkinson’s disease, Coronary heart disease, Hypertension, Cirrhosis, Chronic kidney disease, Hypothyroidism, Diabetes, Neurological complications, Cerebral infarction, Sequelae of cerebral infarction, Neuromuscular disease, Gastroesophageal reflux disease (GERD), Peptic ulcer), surgical operation information (endoscopy type and history of gastrointestinal surgery). POCUS information was acquired by measuring two gastric antrum diameters (D_1_: the craniocaudal diameter, D_2_: the anteroposterior diameter) from serosa to serosa in two positions (supine and right lateral decubitus (RLD) position). VAS score which includes six items (including thirst, hunger, mouth dryness, nausea, vomiting, and fatigue score) and each item is scored on an 11-point scale (range 0–10, scores closer to 10 indicating the patient is more distressed) were collected to evaluate the psychological influence of the oral carbohydrate of the patients before GIE procedures.

### Outcome

Observing gastric contents under direct gastroscopic visualization, we hypothesized that there are two states of outcome: when the gastric contents contain solids or the gastric fluid volume aspirated by gastroscopy ≥0.4 ml·kg^−1^, the patient is classified as full stomach, otherwise, it is considered as empty stomach. In this study, the outcome was positive when the patient was full stomach, otherwise, the outcome was negative when the patient was empty stomach.

### POCUS, anesthesia method and GIE procedure

When patients enter the preoperative preparation room, patients need to accept an open right-upper limb venous pathway. An ultrasound device (SonoSite Edge) fitted with an abdominal probe (2–5 MHz) was used for the POCUS. Before the anesthesia administration, POCUS would be conducted by a trained, experienced sonographer following a standardized gastric scanning protocol. Each patient was scanned in the supine position followed by the RLD position. In both positions, three still images of the antrum were got at rest (between peristaltic contractions). And scanned items include 3-point antrum grade [10] and two antrum diameters (D_1_ and D_2_). Patients were classified as follows: grade 0: the antrum appeared empty on both supine and right lateral decubitus positions; grade 1: gastric fluid was visible on the right lateral decubitus position only, suggesting a small to moderate fluid volume; and grade 2: gastric fluid was observed in the antrum in both supine and right lateral decubitus, suggesting a larger fluid volume. Only patients with grade 0 or 1 antrum can be directly performed the GIE procedure, while those with grade 2 antrum should continue waiting.

In the endoscopy room, patients who satisfied the GIE procedure were transitioned from the RLD position to the left lateral decubitus position, connected to an anesthesia monitor to monitor pulse oximeter oxygen saturation (SpO_2_), electrocardiogram, and blood pressure (BP) of the left upper limb. Patients were given nasal catheter oxygen inhalation, maintaining an oxygen flow rate of 3–5 L min^−1^ for 3 min. In the process of anesthesia induction, patients received an intravenous injection of remifentanil 0.2 μg kg^−1^, then an intravenous injection of propofol 1–2mg kg^−1^ until the MOAA/S score ≤2. The gastric fluid volume was suctioned under gastroscopic vision, measured accurately to ml, and recorded. If patients in this process occur body movement, it is need to append propofol of 30–50mg under the GIE procedure. After the GIE procedure, patients were transferred to the post-anesthesia care unit when their MOAA/S score was ≥4.

### Data processing

First, to prevent the data leakage problem, we divided the control group into a training cohort and a testing cohort with a ratio of 7:3. In this study, ‘data leakage problem’ referred to a narrow ‘data leakage problem’, particularly referring to the problem of ‘train-test contamination’. For example, due to incorrect handling of the relationship between training and testing data in the data preprocessing, the model showed over-optimistic performance in the model validation but showed poor generalization ability when used for new datasets [27,28].

Training cohort was used to construct prediction models and tune hyper-parameters. And the testing cohort was used to verify the generalization of the model [27,28]. Second, we excluded the variables with more than 25% missing data and those samples without POCUS data. Then, K-Nearest Neighbor (KNN) was used to impute the missing continuous variables, and mode imputation was applied to impute the missing categorical variables. Next, univariable analysis was performed to select the variables associated with full stomach (*p-*value <0.05). These variables associated with full stomach would be entered into multivariable logistical regression, then those significant variables would be further entered in the stepwise forward regression analysis for feature selection. One-hot encoding is a preprocessing method that maps non-numerical variables into binary vectors. Moreover, all continuous variables underwent preprocessing for Z-score normalization, and all categorical variables were transformed by One-hot encoding.

### Model development

We constructed two types of ML models including clinical data ML models and POCUS ML models under two positions. For these two types of ML models, we built the following five ML models: logistic regression classifier (LR), random forest classifier (RF), support vector machine classifier (SVC), extreme gradient boosting classifier (XGB), and multiple layer perception (MLP).

With the training cohort, the Grid Search algorithm with 10-fold cross-validation was applied to select the hyper-parameters of each model. 10-fold cross-validation randomly divided the data set into 10 equal parts, nine of which were used to estimate the model, and the remaining was used to evaluate the model. The hyper-parameters of each model were selected to maximize the area under the receiver operating characteristic curve (AUROC). And we used the Delong test to evaluate statistical differences between two AUROCs in these five models. The performance of these models was evaluated by the metrics of discrimination, calibration, and clinical usefulness. The discrimination ability was assessed by AUROC, the area under the precision-recall (PR) curve (AUPRC), accuracy, precision, recall, F1. And AUPRC is more useful for un-balanced datasets to evaluate the performance of binary classifiers than AUROC. F1 score provides a comprehensive evaluation of precision and recall and presents the capture ability of correct positive results. Besides, the indicators of the discrimination ability were ultimately obtained using 10-fold cross-validation calculated the average values of the training and testing sets. The closer these indicators get to 1, the better accuracy the model reaches. The calibration ability was assessed by brier-score. The closer brier-score gets to 0, the better the calibration ability of the model runs. The clinical usefulness of the model was evaluated by decision curve analysis (DCA). Additionally, the You-den index (sensitivity + specificity − 1) determined the optimal cut-off, which enabled the model to have the maximum total predictive ability to detect true positive and negative samples. Accordingly, these metrics also should be verified in the testing cohort at this threshold. Thus, the optimal machine learning model we ultimately selected can accurately predict full stomach in clinical practice.

### Model interpretation

Furthermore, we employed the SHapley Additive exPlanations (SHAP) method to gain insight into the final selected model [29]. The impact degree of the features was explored by visualizing the SHAP values.

### Statistical analysis

First, Shapiro–Wilk Test was used to evaluate the normality of continuous variables. Continuous variables were described as mean ± standard deviation (SD) or median ± interquartile range (IQR), using the student *t*-test or Mann–Whitney *U* test for comparison. And categorical variables were expressed as count (frequency), using the chi-square test or Fisher’s exact test for comparison. A value of *p* < 0.05 was considered significant in all tests.

### Propensity score matching

According to the optimal cut-off, the final model would classify the dataset as empty- and full stomach groups whether the control or the POCs groups. Patients in both empty- and full stomach groups may receive POCs or fasting. Given the differences in the baseline characteristics between the control and POCs groups, propensity score matching (PSM) was performed in both the empty- and full stomach groups to reduce the influence of selection bias caused by covariables, respectively. And PSM was performed based on the stepwise forward regression algorithm result and demographic information using a 1:1 nearest neighbor matching algorithm with a caliper of 0.05. Balance in covariates was evaluated by the standardized mean difference (SMD). An SMD of <0.1 indicates a better balance.

## Results

### Study subjects

**Figure 1.**
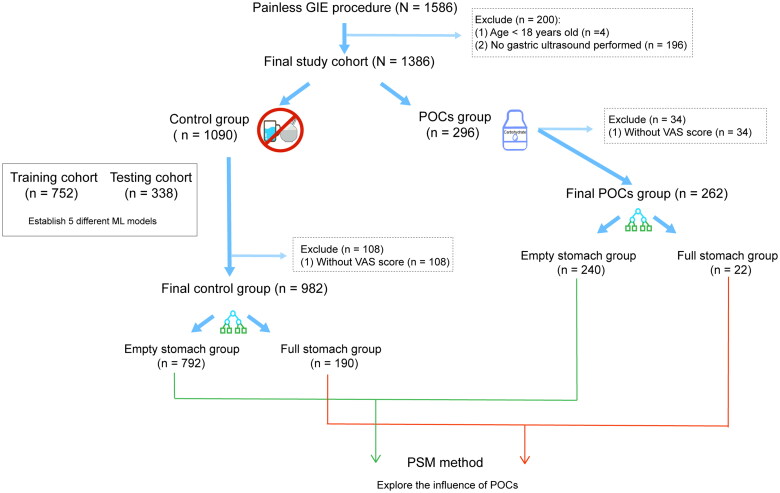
The overall workflow of the entire process is represented as a schematic diagram.

We analyzed data obtained from 1586 consecutive patients with GIE procedures between October 2022 and January 2023 in Nanjing First Hospital, and our study cohort finally consisted of 1386 patients who met the inclusion criteria to establish the ML models. The study process is specifically shown in Figure 1, and this study outcome full stomach was defined as the presence of solid particles and/or the gastric volume >0.4 ml kg^−1^. The median age of our whole sample was 54 yr (42–64yr), and 55.1% were male (Table S1). We divided the whole population into a control group (*n* = 1090) and a POCs group (*n* = 296) based on whether POCs were taken or not. The con­trol group was then divided into the training cohort (70%, *n* = 752) and the testing cohort (30%, *n* = 338). In these two cohorts, the incidence of full stomach was 39.5% (297/752) and 36.7% (124/338) in the training and testing cohorts, respectively. And baseline characteristics of these two cohorts were well-balanced (Table S2). And then, the training cohort was used to construct the ML models and tune the best-fitted hyper-parameters of the model. The testing cohort was used to validate the robustness ability of the ML model.

### Feature selection

**Figure 2.**
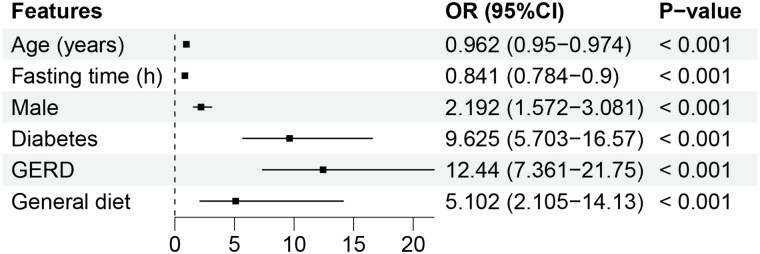
Forest plot of the six significant predictors of full stomach. GERD: gastroesophageal reflux disease.

Our study collected 25 features related to the outcome full stomach. Univariate analysis (Table 1) describes that 7 features were significantly associated with the outcome full stomach. Given that there may be a collinearity problem, we further performed multivariable logistical regression and stepwise forward regression to reduce the redundant information of features. Finally, the six features (Figure 2) were entered to construct the ML models.

**Table 1.** Baseline characteristics of GIE patients in training (*n* = 752) and testing (*n* = 338) cohorts.

Baseline characteristics	Training			Testing	
	Empty-stomach cohort	Full-stomach cohort	*p*-Value	Empty-stomach cohort	Full-stomach cohort
Age (yr), median	56.0 [47.0, 64.0]	53.0 [38.0, 64.0]	<0.001*#	56.0 [46.0, 65.0]	51.0 [37.0, 65.0]
BMI (kg/m^2^), median	24.2 [22.5, 25.5]	23.8 [22.1, 25.5]	0.24#	23.9 [22.1, 25.4]	24.5 [22.2, 26.1]
Drinking, *n* (%)			0.67		
No	370 (81.3)	246 (82.8)		173 (80.8)	104 (83.9)
Yes	85 (18.7)	51 (17.2)		41 (19.2)	20 (16.1)
Smoking, *n* (%)			0.75		
No	395 (86.8)	261 (87.9)		178 (83.2)	109 (87.9)
Yes	60 (13.2)	36 (12.1)		36 (16.8)	15 (12.1)
Sex, *n* (%)			0.001*		
Female	222 (48.8)	109 (36.7)		102 (47.7)	53 (42.7)
Male	233 (51.2)	188 (63.3)		112 (52.3)	71 (57.3)
Comorbid history					
Asthma, *n* (%)			0.07		
No	435 (95.6)	292 (98.3)		206 (96.3)	118 (95.2)
Yes	20 (4.4)	5 (1.7)		8 (3.7)	6 (4.8)
Parkinson disease, *n* (%)			0.84		
No	450 (98.9)	295 (99.3)		209 (97.7)	124 (100.0)
Yes	5 (1.1)	2 (0.7)		5 (2.3)	0 (0.0)
Coronary heart disease, *n* (%)			0.65		
No	425 (93.4)	274 (92.3)		197 (92.1)	108 (87.1)
Yes	30 (6.6)	23 (7.7)		17 (7.9)	16 (12.9)
Hypertension, *n* (%)			0.80		
No	301 (66.2)	200 (67.3)		135 (63.1)	86 (69.4)
Yes	154 (33.8)	97 (32.7)		79 (36.9)	38 (30.6)
Chronic kidney disease, *n* (%)			0.33		
No	428 (94.1)	285 (96.0)		202 (94.4)	120 (96.8)
Yes	27 (5.9)	12 (4.0)		12 (5.6)	4 (3.2)
Cirrhosis, *n* (%)			0.27		
No	440 (96.7)	292 (98.3)		211 (98.6)	123 (99.2)
Yes	15 (3.3)	5 (1.7)		3 (1.4)	1 (0.8)
Hypothyroidism, *n* (%)			0.54		
No	436 (95.8)	288 (97.0)		199 (93.0)	117 (94.4)
Yes	19 (4.2)	9 (3.0)		15 (7.0)	7 (5.6)
Cerebral infarction, *n* (%)			0.93		
No	413 (90.8)	271 (91.2)		195 (91.1)	110 (88.7)
Yes	42 (9.2)	26 (8.8)		19 (8.9)	14 (11.3)
Sequelae of cerebral infarction, *n* (%)			0.91		
No	444 (97.6)	291 (98.0)		210 (98.1)	120 (96.8)
Yes	11 (2.4)	6 (2.0)		4 (1.9)	4 (3.2)
Neuromuscular diseases, *n* (%)			0.63		
No	453 (99.6)	294 (99.0)		213 (99.5)	121 (97.6)
Yes	2 (0.4)	3 (1.0)		1 (0.5)	3 (2.4)
Diabetes, *n* (%)			<0.001*		
No	422 (92.7)	231 (77.8)		202 (94.4)	94 (75.8)
Yes	33 (7.3)	66 (22.2)		12 (5.6)	30 (24.2)
GERD, *n* (%)			<0.001*		
No	436 (95.8)	215 (72.4)		213 (99.5)	90 (72.6)
Yes	19 (4.2)	82 (27.6)		1 (0.5)	34 (27.4)
Endoscopy type, *n* (%)			<0.001*		
Gastric endoscopy	58 (12.7)	119 (40.1)		42 (19.6)	57 (46.0)
Gastrointestinal endoscopy	397 (87.3)	178 (59.9)		172 (80.4)	67 (54.0)
Peptic ulcer, *n* (%)			0.54		
No	443 (97.4)	292 (98.3)		209 (97.7)	121 (97.6)
Yes	12 (2.6)	5 (1.7)		5 (2.3)	3 (2.4)
Preoperative drugs					
PPIs or H_2_RA, *n* (%)			0.77		
No	443 (97.4)	291 (98.0)		209 (97.7)	121 (97.6)
Yes	12 (2.6)	6 (2.0)		5 (2.3)	3 (2.4)
Gastrointestinal motility drugs, *n* (%)			1		
No	445 (97.8)	291 (98.0)		210 (98.1)	122 (98.4)
Yes	10 (2.2)	6 (2.0)		4 (1.9)	2 (1.6)
Gastric mucosal protectant, *n* (%)			0.54		
No	443 (97.4)	292 (98.3)		209 (97.7)	122 (98.4)
Yes	12 (2.6)	5 (1.7)		5 (2.3)	2 (1.6)
Ultrasound data (mm), median					
R_D1	25.6 [23.7, 27.2]	28.1 [25.9, 29.7]	<0.001*#	26.0 [24.1, 27.5]	27.7 [25.1, 30.7]
R_D2	18.5 [16.2, 20.4]	19.7 [17.5, 21.7]	<0.001*#	18.5 [16.2, 20.3]	19.1 [15.8, 21.6]
S_D1	23.7 [19.4, 24.7]	24.5 [21.8, 26.7]	<0.001*#	23.1 [19.0, 24.7]	24.3 [21.5, 26.7]
S_D2	15.3 [13.3, 17.7]	17.5 [15.2, 19.3]	<0.001*#	15.4 [13.3, 17.3]	17.2 [14.3, 19.4]
Fasting profile					
Fasting time (h), median	18.0 [17.0, 19.0]	17.0 [14.0, 19.0]	<0.001*#	18.0 [16.0, 19.0]	15.0 [13.8, 18.00]
No drinking time (h), median	7.0 [6.0, 9.0]	6.0 [6.0, 9.0]	0.16#	7.0 [6.0, 10.0]	7.0 [6.0, 10.0]
Diet, *n* (%)			<0.001*		
Liquid	32 (7.0)	5 (1.7)		14 (6.5)	5 (4.0)
Semi-liquid	369 (81.1)	170 (57.2)		159 (74.3)	55 (44.4)
General diet	54 (11.9)	122 (41.1)		41 (19.2)	64 (51.6)

For continuous variables, data are expressed as the mean ± standard deviation (SD) or median ± interquartile range (IQR). For categorical variables are presented as percentages. *Means included in a multiple logistic regression model. ^#^Means calculated using the Mann–Whitney *U* test.

GIE: gastrointestinal endoscopy; BMI: body mass index; HGB: hemoglobin; GERD: Gastroesophageal reflux disease; PPI or H_2_RA: proton pump inhibitors or H_2_ receptor antagonists; R_D_1_: the longitudinal diameter of right lateral decubitus position; R_D_2_: the anteroposterior diameter of right lateral decubitus position; S_D_1_: the longitudinal diameter of supine position; S_D_2_: the anteroposterior diameter of supine position.

### Model performance

**Figure 3.**
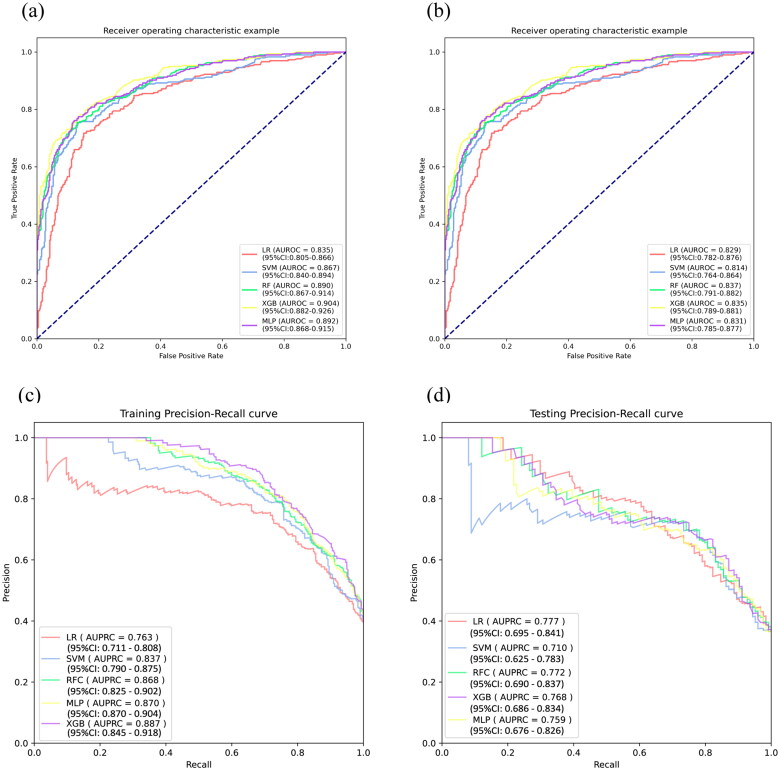
ROC curve and PRC curve for full stomach. ROC curves (a, b) and PRC curves (c, d) for the training and testing cohort, respectively. AUROC: area under the receiver operating characteristic curve; ROC: receive operating characteristic; AUPRC: area under the precision-recall curve; PRC: precision-recall curve.

**Figure 4.**
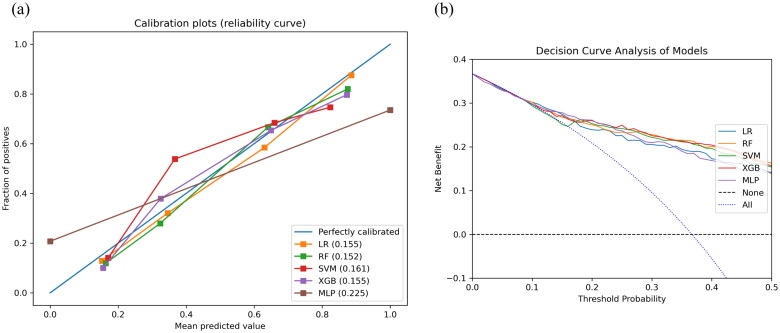
Calibration curve and DCA curve for full stomach. Calibration curve (a) and DCA curve (b) for the testing cohort. DCA: decision curve analysis.

These six features were used to establish the following five ML models: LR, SVM, RF, XGB, and MLP. Table 2 indicated that the RF model has the greatest discriminatory performing ability (the highest AUROC, the second highest F1) among these five ML models in the training cohort. In addition, we validated the robustness ability of those established models according to the determined optimal cut-off in the testing cohort (Table 2), and RF still presented satisfactory performance (Figure 3). The optimal values of the hyper-parameters for each classifier could be specifically viewed in Table S3. A comparison of the AUROC using the Delong test showed no significant differences (Table S4). As for the calibration ability, RF had the lowest Brier score of 15.2% (Figure 4(a)) among all models. In Figure 4(b), the DCA curves revealed no obvious differences among all models. Therefore, we finally selected the RF model representing the clinical ML model to predict the risk of full stomach. For POCUS ML models, the SVM model based on RLD position performs best in this series of POCUS ML models and shows a strong discriminatory ability (Table S5).

**Table 2.** Performance metrics of the five different clinical data ML models in the training and testing cohorts.

		Optimal cut-off	AUROC	AUPRC	Precision %	Recall %	F1 %
LR	Training	0.433	0.835 (0.805–0.866)	0.763 (0.711–0.808)	75.5	71.7	73.6
	Testing		0.829 (0.782–0.876)	0.777 (0.695–0.841)	68.0	68.5	68.3
SVM	Training	0.346	0.867 (0.840–0.894)	0.837 (0.790–0.875)	78.6	75.4	77.0
	Testing		0.814 (0.764–0.864)	0.710 (0.625–0.783)	71.7	73.4	72.5
RF	Training	0.468	0.890 (0.867–0.914)	0.868 (0.825–0.902)	79.2	0.754	77.2
	Testing		0.837 (0.791–0.882)	0.772 (0.690–0.837)	72.1	71.0	71.5
XGB	Training	0.391	0.904 (0.882–0.926)	0.887 (0.845–0.918)	76.9	79.8	78.3
	Testing		0.835 (0.789–0.881)	0.768 (0.686–0.834)	71.5	75.0	73.2
MLP	Training	0.389	0.892 [0.868–0.915]	0.870 (0.827–0.904)	79.0	77.4	78.2
	Testing		0.831 (0.785–0.877)	0.759 (0.676–0.826)	70.5	63.7	66.9

ML: machine learning; LR: logistical regression; SVM: support vector machine; RF: random forest; XGB: extreme gradient boosting; MLP: multiple layer perception.

### Model interpretation

**Figure 5.**
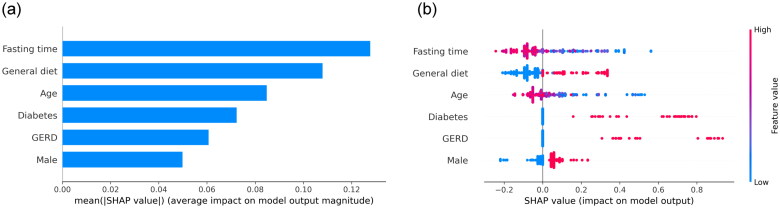
SHAP summary plots of interpretation for the six significant predictors contributing to RF model. Bar chart of the average absolute SHAP value for each significant predictor (a) and dot chart of each significant predictor contributing to RF model. SHAP: SHapley Addictive explanation; RF: random forest.

We calculated the feature importance using the average absolute SHAP values for the RF model, which had the greatest discriminatory ability. Figure 5(a) depicts the top 6 features in descending order, and Figure 5(b) shows an overview of the positive or negative impact of features on the RF model.

### PSM results

RF model divides both the control group (*n* = 982) and the POCs group (*n* = 262) into empty-stomach and full stomach groups at the threshold of 0.468. In the control group, the empty stomach group includes 792 patients, while the full stomach group includes 190 patients. Meanwhile, in the POCs group, the empty stomach group consists of 240 patients, while the full stomach group contains 22 patients. After applying the PSM method according to the items listed in the method section, in the empty stomach group, 229 patients in the POCs group were compared with 229 patients in the control group (Table 3). Table 3 shows the incidence rate of full stomach, Thirst score, Mouth dryness score, and Nausea score of the VAS score item in the POCs group are significantly lower in the POCs group compared with the control group. In the same way, we also applied the PSM method in the full stomach group of both the control group and the oral carbohydrate group. The result (Table 4) reveals that the incidence rate of full stomach and VAS score items except the Thirst score were no significant differences between the control and the POCs groups.

**Table 3.** Demographics and variables included in the RF model before and after PSM in the empty stomach cohort between the control and POCs groups.

	Before PSM				After PSM			
	Control (*n* = 792)	POCs (*n* = 240)	*p*–Value	SMD	Control (*n* = 229)	POCs (*n* = 229)	*p –* Value	SMD
Age (years), median	55.0 [45.0, 64.0]	49.0 [39.8, 59.0]	<0.001	0.392	50.0 [41.0, 59.00]	50.0 [41.0, 60.0]	0.886	0.015
BMI (kg/m^2^), median	24.0 [22.2, 25.4]	24.1 [22.7, 25.6]	0.133	0.123	24.1 [22.3, 25.4]	24.1 [22.7, 25.6]	0.726	0.035
Height (cm), median	164.0 [158.0, 171.0]	165.0 [158.0, 173.0]	0.155	0.126	165.0 [160.0, 173.0]	165.0 [158.0, 172.0]	0.323	0.073
Weight (kg), median	64.0 [58.0, 72.0]	65.0 [59.0, 74.0]	0.067	0.165	65.0 [59.0, 73.0]	65.0 [59.0, 73.0]	0.747	0.024
Fasting time (h), median	18.0 [15.0, 19.0]	18.0 [15.0, 19.0]	0.455	0.09	18.0 [15.0, 19.0]	18.0 [15.0, 19.00]	0.742	0.024
Sex (%)			0.444	0.062			0.639	0.053
Male	450 (56.8)	129 (53.8)			120 (52.4)	126 (55.0)		
Female	342 (43.2)	111 (46.2)			109 (47.6)	103 (45.0)		
Diabetes (%)			0.005	0.276			NA	<0.001
No	763 (96.3)	240 (100.0)			229 (100.0)	229 (100.0)		
Yes	29 (3.7)	0 (0.0)			\	\		
GERD (%)							NA	<0.001
No	792 (100.0)	240 (100.0)	NA	<0.001	229 (100.0)	229 (100.0)		
General food (%)			0.949	0.011				
No	620 (78.3)	189 (78.8)			185 (80.8)	179 (78.2)	0.563	0.065
Yes	172 (21.7)	51 (21.2)			44 (19.2)	50 (21.8)		
Full stomach (%)			0.008	0.209			0.013	0.244
No	574 (72.5)	195 (81.2)			167 (72.9)	190 (83.0)		
Yes	218 (27.5)	45 (18.8)			62 (27.1)	39 (17.0)		
VAS Score								
Thirst	6.0 [5.0, 6.0]	1.0 [1.0, 2.0]	<0.001	3.132	6.0 [4.0, 6.0]	1.0 [1.0, 2.0]	<0.001	3.009
Hunger	6.0 [4.0, 7.0]	6.0 [5.0, 7.0]	<0.001	0.17	5.0 [4.0, 6.0]	6.00 [5.0, 7.0]	<0.001	0.233
Mouth dryness	2.0 [1.0, 3.0]	2.0 [1.0, 3.0]	0.001	0.276	2.0 [1.0, 3.0]	2.0 [1.0, 3.0]	0.047	0.212
Nausea	2.0 [2.0, 3.0]	2.0 [1.0, 2.0]	<0.001	0.741	2.0 [2.0, 3.0]	2.0 [1.0, 2.0]	<0.001	0.754
Vomit	1.0 [0.0, 1.00]	1.0 [0.0, 2.0]	0.093	0.112	1.0 [0.0, 1.0]	1.0 [0.0, 2.0]	0.1	0.147
Fatigue	3.0 [2.0, 3.0]	2.0 [1.0, 3.0]	<0.001	0.273	2.00[2.0, 3.0]	2.0 [1.0, 3.0]	0.091	0.138

RF, random forest; POCs, preoperative oral carbohydrates; GERD, Gastroesophageal reflux disease ; VAS, Visual Analogue Scale.

**Table 4.** Demographics and variables included in the RF model before and after PSM in the high-risk cohort between the control and oral carbohydrate groups.

	Before PSM				After PSM			
	Control (*n* = 190)	POCs (*n* = 22)	*p* Value	SMD	Control (*n* = 14)	POCs (*n* = 14)	*p* Value	SMD
Age (years), median	54.0 [39.0, 65.0]	32.5 [25.8, 44.8]	<0.001	1.01	31.0 [27.0, 40.8]	39.5 [29.0, 47.3]	0.15	0.56
BMI (kg/m^2^), median	24.4 [22.1, 26.3]	24.4 [21.6, 25.7]	0.33	0.29	26.3 [24.3, 27.0]	25.1 [22.8, 26.5]	0.23	0.42
Height (cm), median	165.0 [158.0, 173.0]	163.0 [160.0, 174.0]	0.58	0.09	173.0 [169.3, 174.8]	166.5 [161.0, 177.0]	0.70	0.20
Weight (kg), median	65.0 [59.0, 77.0]	63.5 [55.5, 75.0]	0.63	0.14	78.5 [71.0, 80.0]	72.0 [62.0, 79.0]	0.37	0.40
Fasting time (h), median	18.0 [14.0, 19.0]	16.0 [13.3, 18.0]	0.17	0.24	17.0 [14.0, 18.8]	16.5 [13.8, 18.0]	0.78	0.03
Sex (%)			0.98	0.06			0.42	0.47
Male	107 (56.3)	13 (59.1)			3 (21.4)	6 (42.9)		
Female	83 (43.7)	9 (40.9)			11 (78.6)	8 (57.1)		
Diabetes (%)			<0.001	1.26			NA	<0.001
No	106 (55.8)	22 (100.0)			14 (100.0)	14 (100.0)		
Yes	84 (44.2)	0 (0.0)			\	\		
GERD (%)			<0.001	1.17			NA	<0.001
No	77 (40.5)	0 (0.0)			\	\		
Yes	113 (59.5)	22 (100.0)			14 (100.0)	14 (100.0)		
General food (%)			0.10	0.45			1	0.14
No	98 (51.6)	16 (72.7)			7 (50.0)	8 (57.1)		
Yes	92 (48.4)	6 (27.3)			7 (50.0)	6 (42.9)		
Full stomach (%)			0.45	0.22			0.21	0.67
No	42 (22.1)	7 (31.8)			2 (14.3)	6 (42.9)		
Yes	148 (77.9)	15 (68.2)			12 (85.7)	8 (57.1)		
*VAS Score*								
Thirst	6.0 [4.0, 7.0]	1.0 [1.0, 1.0]	<0.001	3.45	5.5 [3.0, 6.0]	1.0 [1.0, 1.0]	<0.001	2.54
Hunger	6.0 [4.0, 7.0]	6.0 [4.0, 6.0]	0.32	0.24	5.0 [2.0, 7.0]	6.0 [4.5, 6.8]	0.74	0.19
Mouth dryness	3.00 [2.0, 3.0]	1.0 [1.0, 2.0]	<0.001	0.84	1.0 [1.0, 3.0]	1.0 [1.0, 3.0]	0.96	0.05
Nausea	2.0 [1.0, 3.0]	1.0 [1.0, 2.0]	<0.001	0.94	2.0 [0.3, 3.0]	1.0 [1.0, 2.0]	0.27	0.46
Vomit	1.0 [0.3, 2.0]	1.0 [0.0, 1.0]	0.01	0.65	1.0 [0.3, 2.0]	1.0 [0.3, 1.0]	0.33	0.37
Fatigue	2.0 [1.0, 3.0]	2.0 [1.0, 3.0]	0.54	0.11	1.5 [1.0, 2.0]	2.0 [1.3, 3.0]	0.14	0.58

RF: random forest; POCs: preoperative oral carbohydrates; GERD: gastroesophageal reflux disease; VAS: Visual Analogue Scale.

### Construct the web calculator

**Figure 6.**
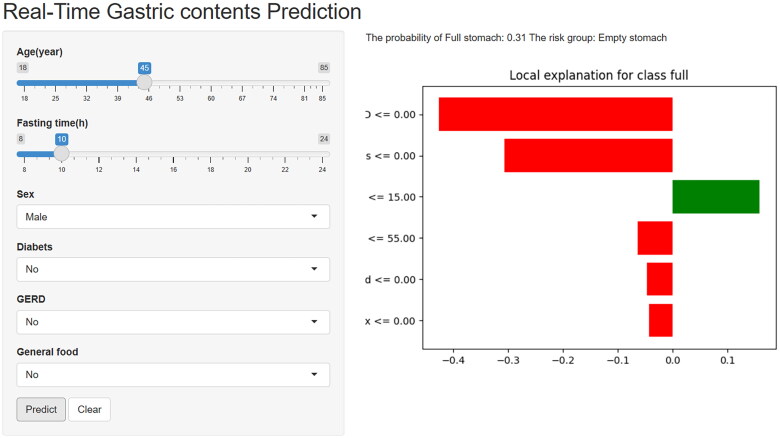
The web calculator was constructed based on RF model. RF: random forest.

To facilitate the promotion and utilization in clinical practice, we further deployed the RF model on the web. Researchers and clinicians can visit the website (https://medication.shinyapps.io/RF_model/), and fill in relevant patient information in several seconds. They will obtain a corresponding risk stratification result and a lime plot explaining the degree of each feature contributes to the overall outcome of each patient (Figure 6).

## Discussion

Considering that an efficient, inexpensive, and accurate method is clinically needed to assess the full stomach, we built the ML models in this study. Five ML algorithms performed well for both the clinical data ML models and the POCUS ML models including antrum diameters, with an average AUROC of 0.829 and 0.903, respectively. Among the five clinical data ML models, RF was the best performer. The test cohort AUROC was 0.837 and had good precision and recall with an F1 value of 71.5%, showing a low level of risk overestimation and less leakage, which was consistent with the calibration curve. The developed RF model predicted full stomach with good accuracy and interpretability, and the more convenient clinical data ML model is recommended in patient-rich outpatient clinics, primary hospitals, remote regions, and specific clinical settings where POCUS is not available.

The established RF model showed good predictive performance and was reliable. This model had a similar AUROC to the POCUS-based linear model (AUROC of 0.84) proposed by Bouvet and colleagues [8] for the same outcome (solid particles and/or fluid volume >0.4 ml kg^−1^), which showed that the performance of ML model with clinical data only is comparable to the POCUS-based linear model when semi- quantifying gastric fluid volume. In addition, our study has several advantages compared to the previous traditional linear analysis method. First, the dataset is split into training and validation cohorts to prevent overfitting, ensuring that the model can be generalized beyond the specific patient cohort used to train this algorithm. This method has not been used in previous linear regression studies. Second, multiple risk factors affecting gastric contents were investigated, which we believe more accurately reflects the realities of anesthesiologists rapidly assessing real-world patient cohorts when POCUS is absent, as complex medical histories and clinical characteristics can affect overall patient outcomes. Third, the RF is an excellent integrated learning algorithm consisting of many decision trees (base evaluators) that are all independent. RF averages the predictions of the base evaluators or uses the majority voting principle to determine the outcome and thus has better classification performance than individual evaluators [30–32].

The RF model is convenient to use and has excellent clinical applicability. Although the POCUS ML model had the highest AUROC, POCUS is not routine in elective GIE outpatients, which requires extra time and cost to use the model. In contrast, the clinical data ML model avoided POCUS and the variables included were routine and easily available, which allowed the RF model to be conveniently and efficiently applied with an acceptable reduced performance. In addition, based on the RF algorithm model, an online accessible web calculator was created to facilitate clinical use. As the variables in the RF model were readily available, the web calculator can be integrated with hospital electronic patient records to form a supported prediction system. In practice, the supported prediction system would access the patients’ clinical information and feed the required variables to the web calculator, automatically calculating the patient’s risk of a full stomach. Moreover, based on each patient’s actual profile, waterfall plots were provided to explain the contribution of each variable to the overall outcome, which increased the interpretability of the model and provided more information to the anesthesiologists.

The model can assist anesthesiologists in guiding patients in the use of POCs. The American Society of Anesthesiologists (ASA) strongly recommends that healthy infants, children, and adults who have oral clear liquids 2 h before surgery should maintain anesthesia and sedation, and POCs can alleviate discomfort due to long fasting [23,24]. According to our findings, POCs did not increase the stomach risk in full stomach patients, as demonstrated in several non-inferiority studies [20,33,34]. Second, the stomach risk was lower in empty stomach POCs patients than in empty stomachs with complete fasting. Some studies had similar findings, especially in pediatric patients, for whom preoperative 1h carbohydrate has been proposed [22,35–37]. Third, comfort was variously improved in empty stomach and full stomach patients after POCs, and the improvement was more obvious in the empty group, probably because empty stomach patients had more rapid gastric emptying with earlier and more intense discomfort during fasting. These model-based analysis results may provide information for anesthesiologists to guide patients on POCs.

Multivariate logistic regression determined six independent risk factors to predict full stomach. Among them, age, male, diabetes, and GERD have been reported in some studies [16,38]. In our dataset, fasting time and general diet were significant predictors, rarely mentioned in fasted patients. In a survey of pediatric patients, each hour of fasting was associated with lower odds of full stomach (OR = 0.79) [39]. Some studies also found that despite fasting, some adult patients were still full [7,8]. This indicates that for patients with delayed gastric emptying, an 8-h fasting time may not be sufficient. It has been shown that the general diet empties more slowly than liquid or semi-fluid foods [40], and we found that the gastric fluid volume still differed between patients with different diets after fasting up to 8h. This is correlated with the patient’s actual gastrointestinal preparation, who are usually told to eat a liquid or semi-liquid diet the night before. Based on this, the model may help anesthesiologists guide patients’ diets by quantifying the contribution of a general diet to full stomach, which requires further study.

There are some limitations of this study. First, our dataset emerged from a single organization, and the generalization of the best-performing model needs to be tested in other centers. The web calculator we provided may help address this, as researchers worldwide can access and validate it. Second, diabetic patients were not loaded with POCs due to elevated glycemic risk, and we could not explore the risks and benefits of POCs in this population. Diabetic patients exceed 15% of surgical patients [41], whose perioperative gastrointestinal preparation deserves attention. There has been some evidence that POCs loading may be safe and effective in diabetic patients [42,43], but most studies assessing the efficacy of POCs excluded them. Finally, the model was developed in GIE patients, and its suitability for nonoperating room anesthesia patients other than GIE (e.g. bronchoscopy) and operating room patients is unknown, which requires further study.

## Conclusions

In conclusion, this study demonstrated the potential of the ML model in predicting the full stomach in patients undergoing elective GIE. The model and its web calculator should be used in specific settings where demand efficiency or POCUS is unavailable to enhance clinical judgment and support daily decision-making. Analysis of POCs based on model outcomes showed that POCs did not increase the stomach risk in full stomach patients, they even decrease stomach risk in empty stomach patients. Both groups had improved comfort, and the benefit may be greater in empty stomach group. These findings may aid the anesthesiologist in precise the patients’ gastrointestinal preparation using the model.

## Supplementary Material

Supplemental Material

## Data Availability

The data that support the findings of this study are available from the corresponding author upon reasonable request.
