# Supplementary material for: A convenient machine learning model to predict full stomach and evaluate the safety and comfort improvements of preoperative oral carbohydrate in patients undergoing elective painless gastrointestinal endoscopy
Source: Ann Med. 2023 Dec 18;55(2):2292778. doi: 10.1080/07853890.2023.2292778 (PMC10732178; doi:10.1080/07853890.2023.2292778)
Supplement: Supplemental Material [file IANN_A_2292778_SM0028.docx]

***Supplemental Material***

**A convenient machine learning model to predict full stomach and** **evaluate the safety and comfort** **improvements of preoperative oral carbohydrate in patients undergoing elective** **painless gastrointestinal endoscopy**

Yuzhan Jin ^a, b*^, Mingtao Ma ^c, d*^, Yuqing Yan ^a, b*^, Yaoyi Guo ^c^, Yue Feng ^c^, Chen Chen ^b, e^, Yi Zhong ^c^, Kaizong Huang ^b, e^, Yanna Si ^c^ and Jianjun Zou ^b, e^

**CONTACT** Yanna Si [siyanna@163.com](mailto:siyanna@163.com) Department of Anesthesiology, Nanjing First Hospital, Nanjing Medical University, Nanjing 210006, China; Jianjun Zou [zoujianjun100@126.com](mailto:zoujianjun100@126.com) Department of Clinical Pharmacology, Nanjing First Hospital, Nanjing Medical University, Nanjing 210006, China.

1. **Supplemental Tables**

**Table S1** Baseline characteristics of the whole GIE population (n =1386).

| **Baseline characteristics** | **All** | **Empty stomach cohort** | **Full stomach cohort** |
| --- | --- | --- | --- |
| Age, median (IQR) | 54.0 [42.0, 64.0] | 55.0 [45.0, 64.0] | 48.5 [35.0, 63.0] |
| BMI, median (IQR) | 24.1 [22.3, 25.5] | 24.2 [22.5, 25.5] | 23.9 [22.1, 25.5] |
| Drinking, n (%) |  |  |  |
| No | 1119 (80.7) | 723 (80.5) | 396 (81.1) |
| Yes | 267 (19.3) | 175 (19.5) | 92 (18.9) |
| Smoking, n (%) |  |  |  |
| No | 1174 (84.7) | 756 (84.2) | 418 (85.7) |
| Yes | 212 (15.3) | 142 (15.8) | 70 (14.3) |
| Sex, n (%) |  |  |  |
| Female | 622 (44.9) | 432 (48.1) | 190 (38.9) |
| Male | 764 (55.1) | 466 (51.9) | 298 (61.1) |
| *Comorbid history* |  |  |  |
| Asthma, n (%) |  |  |  |
| No | 1330 (96.0) | 859 (95.7) | 471 (96.5) |
| Yes | 56 (4.0) | 39 (4.3) | 17 (3.5) |
| Parkinson disease, n (%) |  |  |  |
| No | 1371 (98.9) | 885 (98.6) | 486 (99.6) |
| Yes | 15 (1.1) | 13 (1.4) | 2 (0.4) |
| Coronary heart disease, n (%) |  |  |  |
| No | 1281 (92.4) | 834 (92.9) | 447 (91.6) |
| Yes | 105 (7.6) | 64 (7.1) | 41 (8.4) |
| Hypertension, n (%) |  |  |  |
| No | 948 (68.4) | 597 (66.5) | 351 (71.9) |
| Yes | 438 (31.6) | 301 (33.5) | 137 (28.1) |
| Chronic kidney disease, n (%) |  |  |  |
| No | 1329 (95.9) | 858 (95.5) | 471 (96.5) |
| Yes | 57 (4.1) | 40 (4.5) | 17 (3.5) |
| Cirrhosis, n (%) |  |  |  |
| No | 1358 (98.0) | 876 (97.6) | 482 (98.8) |
| Yes | 28 (2.0) | 22 (2.4) | 6 (1.2) |
| Hypothyroidism, n (%) |  |  |  |
| No | 1311 (94.6) | 843 (93.9) | 468 (95.9) |
| Yes | 75 (5.4) | 55 (6.1) | 20 (4.1) |
| Cerebral infarction, n (%) |  |  |  |
| No | 1264 (91.2) | 817 (91.0) | 447 (91.6) |
| Yes | 122 (8.8) | 81 (9.0) | 41 (8.4) |
| Sequelae of cerebral infarction, n (%) | |  |  |
| No | 1358 (98.0) | 880 (98.0) | 478 (98.0) |
| Yes | 28 (2.0) | 18 (2.0) | 10 (2.0) |
| Neuromuscular Diseases, n (%) | |  |  |
| No | 1375 (99.2) | 893 (99.4) | 482 (98.8) |
| Yes | 11 (0.8) | 5 (0.6) | 6 (1.2) |
| Diabetes, n (%) |  |  |  |
| No | 1245 (89.8) | 853 (95.0) | 392 (80.3) |
| Yes | 141 (10.2) | 45 (5.0) | 96 (19.7) |
| GERD, n (%) |  |  |  |
| No | 1226 (88.5) | 870 (96.9) | 356 (73.0) |
| Yes | 160 (11.5) | 28 (3.1) | 132 (27.0) |
| Peptic ulcer, n (%) |  |  |  |
| No | 1347 (97.2) | 871 (97.0) | 476 (97.5) |
| Yes | 39 (2.8) | 27 (3.0) | 12 (2.5) |
| *Preoperative drugs* |  |  |  |
| PPIs or H_2_RA, n (%) |  |  |  |
| No | 1348 (97.3) | 872 (97.1) | 476 (97.5) |
| Yes | 38 (2.7) | 26 (2.9) | 12 (2.5) |
| Gastrointestinal motility drugs, n (%) | |  |  |
| No | 1359 (98.1) | 880 (98.0) | 479 (98.2) |
| Yes | 27 (1.9) | 18 (2.0) | 9 (1.8) |
| Gastric mucosal protectant, n (%) | |  |  |
| No | 1350 (97.4) | 874 (97.3) | 476 (97.5) |
| Yes | 36 (2.6) | 24 (2.7) | 12 (2.5) |
| Ultrasound data (mm), median |  |  |  |
| R_D1 | 25.9 [23.9, 28.2] | 25.6 [23.6, 27.1] | 27.6 [25.1, 29.6] |
| R_D2 | 18.6 [15.7, 20.6] | 18.4 [15.6, 20.1] | 19.3 [16.3, 21.5] |
| S_D1 | 23.6 [19.4, 24.9] | 23.3 [18.6, 24.7] | 24.1 [20.8, 26.3] |
| S_D2 | 15.6 [13.4, 18.3] | 15.2 [13.1, 17.4] | 17.1 [14.3, 19.2] |
| Fasting profile |  |  |  |
| Fasting time (h), median (IQR) | 18.0 [15.0, 19.0] | 18.0 [16.0, 19.0] | 16.0 [14.0, 18.0] |
| No drinking time (h), median (IQR) | 6.0 [5.0, 8.0] | 6.0 [3.0, 8.0] | 6.0 [5.0, 9.0] |
| Diet (%) |  |  |  |
| Liquid | 80 (5.8) | 63 (7.0) | 17 (3.5) |
| Semi-liquid | 960 (69.3) | 694 (77.3) | 266 (54.5) |
| General diet | 346 (25.0) | 141 (15.7) | 205 (42.0) |

GIE, gastrointestinal endoscopy; BMI, body mass index; HGB, hemoglobin; GERD, Gastroesophageal reflux disease; PPI or H_2_RA, Proton Pump Inhibitors or H_2_ receptor antagonists; R_D1, the longitudinal diameter of right lateral decubitus position; R_D2, the anteroposterior diameter of right lateral decubitus position; S_D1, the longitudinal diameter of supine position; S_D2, the anteroposterior diameter of supine position.

**Table S2** The difference in baseline characteristics of patients in training (n = 752) and testing cohort (n = 338).

|  | **Training cohort** | **Testing cohort** | ***P*-value** |
| --- | --- | --- | --- |
| Age (years), median | 55.0 [43.8, 64.0] | 55.0 [44.0, 65.0] | 0.47 |
| BMI (kg/m^2^), median | 24.1 [22.3, 25.5] | 24.15 [22.1, 25.6] | 0.83 |
| Drinking |  |  | 1 |
| No | 616 (81.9) | 277 (82.0) |  |
| Yes | 136 (18.1) | 61 (18.0) |  |
| Smoking |  |  | 0.35 |
| No | 656 (87.2) | 287 (84.9) |  |
| Yes | 96 (12.8) | 51 (15.1) |  |
| Sex |  |  | 0.62 |
| Female | 331 (44.0) | 155 (45.9) |  |
| Male | 421 (56.0) | 183 (54.1) |  |
| Comorbid history |  |  |  |
| Asthma (%) |  |  | 0.62 |
| No | 727 (96.7) | 324 (95.9) |  |
| Yes | 25 (3.3) | 14 (4.1) |  |
| Parkinson disease (%) |  |  | 0.63 |
| No | 745 (99.1) | 333 (98.5) |  |
| Yes | 7 (0.9) | 5 (1.5) |  |
| Coronary heart disease (%) |  |  | 0.16 |
| No | 699 (93.0) | 305 (90.2) |  |
| Yes | 53 (7.0) | 33 (9.8) |  |
| Hypertension (%) |  |  | 0.74 |
| No | 501 (66.6) | 221 (65.4) |  |
| Yes | 251 (33.4) | 117 (34.6) |  |
| Chronic kidney disease (%) |  |  | 0.87 |
| No | 713 (94.8) | 322 (95.3) |  |
| Yes | 39 (5.2) | 16 (4.7) |  |
| Cirrhosis (%) |  |  | 0.19 |
| No | 732 (97.3) | 334 (98.8) |  |
| Yes | 20 (2.7) | 4 (1.2) |  |
| Hypothyroidism (%) |  |  | 0.06 |
| No | 724 (96.3) | 316 (93.5) |  |
| Yes | 28 (3.7) | 22 (6.5) |  |
| Cerebral infarction (%) |  |  | 0.79 |
| No | 684 (91.0) | 305 (90.2) |  |
| Yes | 68 (9.0) | 33 (9.8) |  |
| Sequelae of cerebral infarction (%) |  |  | 1 |
| No | 735 (97.7) | 330 (97.6) |  |
| Yes | 17 (2.3) | 8 (2.4) |  |
| Neuromuscular Diseases (%) |  |  | 0.61 |
| No | 747 (99.3) | 334 (98.8) |  |
| Yes | 5 (0.7) | 4 (1.2) |  |
| Diabetes (%) |  |  | 0.81 |
| No | 653 (86.8) | 296 (87.6) |  |
| Yes | 99 (13.2) | 42 (12.4) |  |
| GERD (%) |  |  | 0.19 |
| No | 651 (86.6) | 303 (89.6) |  |
| Yes | 101 (13.4) | 35 (10.4) |  |
| Peptic ulcer (%) |  |  | 1 |
| No | 735 (97.7) | 330 (97.6) |  |
| Yes | 17 (2.3) | 8 (2.4) |  |
| Preoperative drugs |  |  |  |
| PPIs or H_2_RA (%) |  |  | 1 |
| No | 734 (97.6) | 330 (97.6) |  |
| Yes | 18 (2.4) | 8 (2.4) |  |
| Gastrointestinal motility drugs (%) |  |  | 0.88 |
| No | 736 (97.9) | 332 (98.2) |  |
| Yes | 16 (2.1) | 6 (1.8) |  |
| Gastric mucosal protectant (%) |  |  | 1 |
| No | 735 (97.7) | 331 (97.9) |  |
| Yes | 17 (2.3) | 7 (2.1) |  |
| Ultrasound data (mm), median |  |  |  |
| R_D1 | 26.5 [24.3, 28.4] | 26.4 [24.3, 28.48] | 0.73 |
| R_D2 | 19.2 [16.5, 20.7] | 18.6 [16.2, 20.6] | 0.08 |
| S_D1 | 23.8 [20.2, 25.5] | 23.7 [19.6, 25.3] | 0.44 |
| S_D2 | 16.1 [13.8, 18.6] | 15.8 [13.5, 18.4] | 0.22 |
| Ultrasound to GIE time (min), median | 6.0 [5.0, 6.0] | 6.0 [5.25, 6.0] | 0.07 |
| *Fasting profile* |  |  |  |
| Fasting time (h), median | 18.0 [15.0, 19.0] | 18.00 [15.0, 19.0] | 0.11 |
| No drinking time (h), median | 7.0 [6.0, 9.0] | 7.00 [6.0, 10.0] | 0.20 |
| Diet (%) |  |  | 0.02 |
| Liquid | 37 (4.9) | 19 (5.6) |  |
| Semi-liquid | 539 (71.7) | 214 (63.3) |  |
| General food | 176 (23.4) | 105 (31.1) |  |
| Full stomach |  |  | 0.42 |
| No | 455 (60.5) | 214 (63.3) |  |
| Yes | 297 (39.5) | 124 (36.7) |  |

GIE, gastrointestinal endoscopy; BMI, body mass index; HGB, hemoglobin; GERD, Gastroesophageal reflux disease; PPI or H_2_RA, Proton Pump Inhibitors or H_2_ receptor antagonists; R_D1, the longitudinal diameter of right lateral decubitus position; R_D2, the anteroposterior diameter of right lateral decubitus position; S_D1, the longitudinal diameter of supine position; S_D2, the anteroposterior diameter of supine position.

**Table S3** The best hyper-parameters for the five clinical data ML models.

| **Classifier models** | **Hyper parameters** | **Optimal value** |
| --- | --- | --- |
| LR | C | 1 |
|  | Penalty | L2 |
|  | Solver | liblinear |
| SVM | C | 0.8 |
|  | Gamma | 0.5 |
|  | Kernel | rbf |
| RF | N-estimators | 500 |
|  | Max-depth | 6 |
|  | Min-sample leaf | 4 |
|  | Min-sample split | 4 |
| XGB | N-estimators | 300 |
|  | Max-depth | 5 |
|  | gamma | 0.3 |
|  | Leaning-rate | 0.01 |
|  | Colsample-bytree | 1 |
| MLP | Activation | tanh |
|  | Hidden-layer-size | (100,) |
|  | Leaning-rate | invscaling |
|  | learning_rate_init | 0.01 |

LR, logistic regression; SVM, support vector machine; RF, random forest; XGB, extreme gradient boosting; MLP, multilayer perceptron.

**Table S4** Delong test result of between RF model and the other ML models.

|  | P-value |
| --- | --- |
| RF-LR | 0.4421 |
| RF-SVM | 0.0514 |
| RF-XGB | 0.7179 |
| RF-MLP | 0.5771 |

RF, random forest; ML, machine learning; LR, logistic regression; SVM, support vector machine; XGB, extreme gradient boosting; MLP, multilayer perceptron.

**Table S5** Performance metrics of the five different POCUS ML models under Sup and RLD positions, respectively in the testing cohort.

|  |  | **Optimal cut-off** | **AUROC** | **AUPRC %**  **(95% CI)** | **Precision %** | **Recall %** | **F1 %** |
| --- | --- | --- | --- | --- | --- | --- | --- |
| LR | Sup | 0.453 | 0.895  (0.858-0.931) | 85.4  (78.1-90.6) | 74.6 | 71.0 | 72.7 |
|  | RLD | 0.396 | 0.886  (0.848-0.924) | 84.1  (76.6-89.6) | 73.7 | 79.0 | 76.3 |
| SVM | Sup | 0.319 | 0.908  (0.876-0.940) | 85.9  (78.6-91.0) | 67.3 | 89.5 | 76.8 |
|  | RLD | 0.372 | 0.915  (0.884-0.945) | 85.9  (78.6-91.0) | 75.2 | 83.1 | 78.9 |
| RF | Sup | 0.428 | 0.900  (0.867-0.932) | 85.0  (77.6-90.3) | 74.6 | 80.6 | 77.5 |
|  | RLD | 0.416 | 0.917  (0.888-0.946) | 86.6  (79.4-91.5) | 76.1 | 82.3 | 79.1 |
| XGB | Sup | 0.436 | 0.906  (0.875-0.937) | 86.0  (78.8-91.1) | 73.7 | 79.0 | 76.3 |
|  | RLD | 0.388 | 0.902  (0.869-0.934) | 84.7  (77.2-90.0） | 73.9 | 79.8 | 76.7 |
| MLP | Sup | 0.526 | 0.876  (0.840-0.913) | 81.6  （73.8-87.5) | 67.9 | 73.4 | 70.5 |
|  | RLD | 0.422 | 0.895  (0.861-0.929) | 84.5  (77.0-89.9) | 72.6 | 79.0 | 75.7 |

ML, machine learning; Sup, supine; RLD, right lateral decubitus; LR, logistical regression; SVM, support vector machine; RF, random forest; XGB, extreme gradient boosting; MLP, multiple layer perception.

1. **Supplementary Figures**

None.
